# Anaesthetic Management of Neurofibromatosis of an Unknown Subtype for Emergency Caesarean Section

**DOI:** 10.7759/cureus.104539

**Published:** 2026-03-02

**Authors:** Stephanie Shirto, Ryan Glaser

**Affiliations:** 1 Anaesthesiology, University of the Witwatersrand, Johannesburg, ZAF

**Keywords:** cesarean section, emergency caesarean section, neuraxial anesthesia, neurofibromatosis, obstetric anesthesia, pregnancy complications, risk assessment, spinal anesthesia

## Abstract

Neurofibromatosis is a heterogeneous group of inherited neurocutaneous disorders with variable multisystem involvement and important implications for obstetric anaesthesia. Anaesthetic concerns include potential airway involvement, occult spinal or intracranial tumours, cardiovascular instability, and the association with catecholamine-secreting tumours. These factors often lead to a preference for general anaesthesia, despite well-recognised maternal risks associated with general anaesthesia in obstetric practice. Decision-making is further complicated when neurofibromatosis is previously undiagnosed and unclassified, and when preoperative imaging is unavailable. We report the case of a 33-year-old gravida 3 para 2 woman at term gestation who required emergency caesarean delivery for fetal distress after declining a trial of vaginal birth following a previous caesarean section. She had extensive cutaneous neurofibromas but no prior diagnosis, genetic classification, or radiological evaluation. The subtype of neurofibromatosis was therefore unknown at presentation. Clinical assessment revealed no neurological symptoms, no focal neurological deficits, no features suggestive of raised intracranial pressure, no clinical evidence of airway or mediastinal involvement, and no history suggestive of catecholamine excess. The case occurred in a resource-limited setting, where urgent neuroimaging was not available. Following senior anaesthetic assessment and informed consent, spinal anaesthesia was selected after careful consideration of the relative risks of neuraxial versus general anaesthesia in the context of diagnostic uncertainty and obstetric urgency. Intrathecal hyperbaric bupivacaine with fentanyl was administered, producing an adequate surgical block. Anticipated spinal-induced hypotension was managed with titrated phenylephrine boluses, and haemodynamic stability was maintained throughout the procedure. The caesarean section and postoperative course were uneventful, with no neurological or cardiovascular complications. This case highlights the importance of phenotype-based clinical risk stratification when managing parturients with suspected but unclassified neurofibromatosis. In the absence of neurological deficits or features suggestive of raised intracranial pressure, neuraxial anaesthesia may be a reasonable option even when imaging is unavailable. This approach may be particularly relevant in emergency obstetric scenarios and resource-limited settings, where delays associated with investigation may increase maternal and fetal risk. The case adds to limited evidence supporting judicious use of spinal anaesthesia in selected parturients with suspected neurofibromatosis and underscores the need for individualized clinical judgement rather than routine exclusion of neuraxial techniques.

## Introduction

Neurofibromatosis comprises a group of inherited neurocutaneous disorders characterised by variable involvement of the skin, nervous system, skeleton, cardiovascular system, and endocrine organs. The two most recognised subtypes, neurofibromatosis type 1 (NF1) and neurofibromatosis type 2 (NF2), differ substantially in their clinical manifestations and anaesthetic implications [1-3]. NF1 is more common and typically presents with cutaneous neurofibromas and café-au-lait macules, whereas NF2 is characterised by frequent central nervous system involvement, including spinal tumours and bilateral vestibular schwannomas [2,3]. This distinction is clinically important because neuraxial risk is largely driven by the likelihood of intraspinal tumours, which is far greater in NF2 than NF1 [2,3].

From an anaesthetic perspective, neurofibromatosis presents multiple challenges. Pregnancy is associated with increased maternal and obstetric risk in patients with neurofibromatosis, including higher rates of hypertensive disorders and caesarean delivery [4,5]. These include potential airway involvement from neurofibromas of the tongue, pharynx, or larynx; spinal or intracranial tumours that may complicate neuraxial techniques; cardiovascular instability; and the association with pheochromocytoma [4,6]. Occult spinal tumours, which may increase the risk of neurological injury or altered CSF dynamics during neuraxial anaesthesia. In obstetric patients, these risks must be weighed against the well-established maternal and fetal benefits of neuraxial anaesthesia and the increased morbidity associated with general anaesthesia for caesarean delivery [7]. In obstetrics, general anaesthesia carries higher risks of failed intubation, aspiration, and maternal mortality compared with neuraxial techniques.

Pregnancy may further complicate neurofibromatosis, with reports of increased size and number of neurofibromas and higher rates of obstetric complications, including hypertensive disorders and caesarean delivery [3,5]. Despite this, many parturients with neurofibromatosis remain neurologically asymptomatic, and clinically significant central nervous system involvement is uncommon, particularly in NF1 [2,3].

Anaesthetic decision-making becomes especially complex when neurofibromatosis is previously undiagnosed, the subtype is unconfirmed, and preoperative imaging is unavailable. This scenario is not uncommon in emergency obstetric and resource-limited settings. However, the literature provides little practical guidance when neurofibromatosis is suspected but unclassified and neuroimaging is unavailable, with most recommendations based on case reports and expert opinion.

Therefore, we present a case of emergency caesarean delivery under spinal anaesthesia in a parturient with clinically suspected but unclassified neurofibromatosis, highlighting a pragmatic, phenotype-based approach to anaesthetic risk assessment.

## Case presentation

A 33-year-old woman, gravida 3 para 2, at 40 weeks’ gestation, presented for an emergency caesarean section due to fetal distress and maternal decision to decline a trial of vaginal birth after a previous caesarean section. Her weight was approximately 70 kg. She had two prior deliveries: one spontaneous vaginal delivery and one caesarean delivery in 2023. She had no other documented surgical history. Family history of neurofibromatosis was unknown.

**Figure 1:**
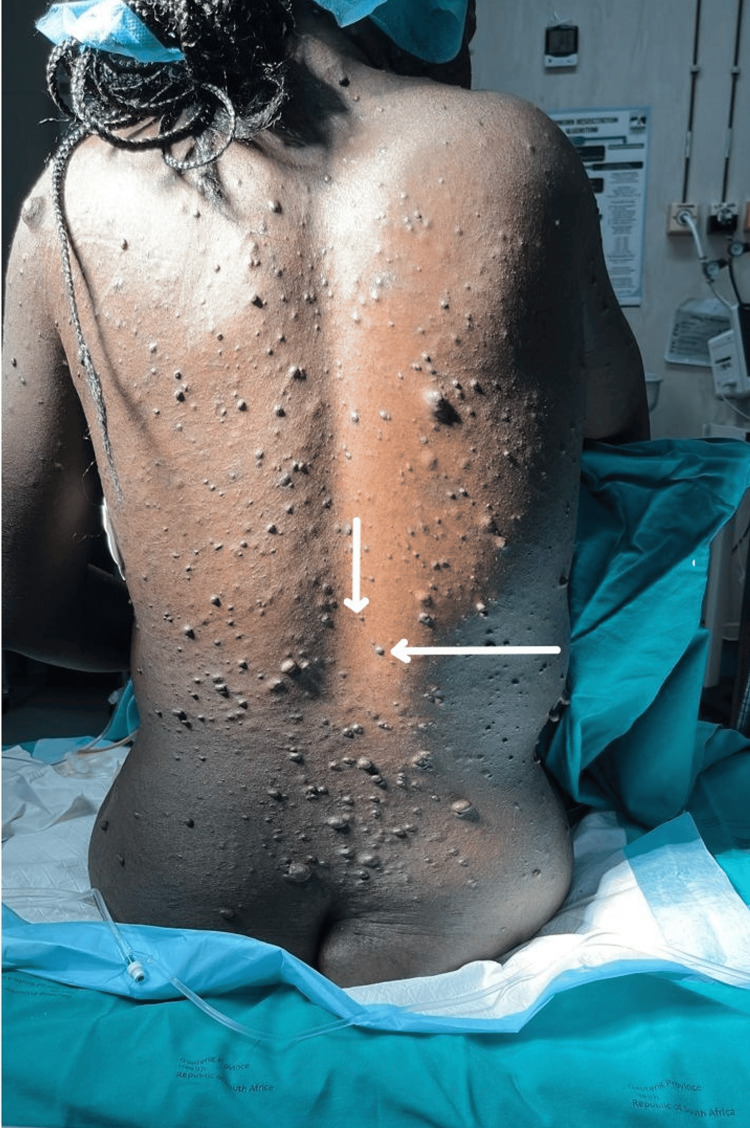
Clinical photograph of the back demonstrating extensive cutaneous neurofibromas and the intended lumbar neuraxial insertion site Clinical photograph demonstrating numerous soft, pedunculated, and sessile cutaneous nodules diffusely distributed over the posterior trunk, consistent with cutaneous neurofibromas. The white arrows indicate the intended L3-L4 interspace selected for neuraxial block placement with least visible and palpable cutaneous nodules. Widespread involvement of the lumbar region highlights practical challenges for neuraxial anaesthesia, including identification of an optimal puncture site and the possibility of underlying intraspinal pathology in a patient with previously undiagnosed neurofibromatosis.

The patient presented with a clinical diagnosis of neurofibromatosis type unspecified, characterised by widespread cutaneous neurofibromas involving the trunk and limbs, as shown in Figure 1. She had no known central nervous system involvement and no previous neurological investigations. There was no history of seizures, focal neurological deficits, radicular pain, sphincter disturbance, or chronic headaches. She denied symptoms suggestive of raised intracranial pressure, including persistent headaches, visual disturbances, nausea, or vomiting.

Cardiovascular examination was normal, with no hypertension recorded during antenatal care. There was no history of episodic headaches, palpitations, diaphoresis, or labile blood pressure to suggest pheochromocytoma. Respiratory examination was unremarkable, with no clinical features of mediastinal masses or restrictive lung disease. Airway assessment revealed Mallampati class I, normal mouth opening, normal neck mobility, and no visible or palpable airway neurofibromas. There were no clinical features suggestive of laryngeal or oropharyngeal involvement. The absence of features like hearing loss and neurological symptoms made NF2 less likely, although definitive subtyping was not possible without imaging/genetic testing. In summary, this was a neurologically asymptomatic parturient with extensive cutaneous neurofibromas and no clinical features of CNS, airway, or endocrine involvement.

Given the emergency nature of the procedure, no preoperative imaging was available. The patient was assessed by the anaesthesia team, and the risks and benefits of neuraxial versus general anaesthesia were discussed. Particular emphasis was placed on the potential risks of general anaesthesia, including airway difficulty, aspiration risk, and haemodynamic instability, as well as the theoretical risks of neuraxial anaesthesia in NF1. After informed consent, a decision was made to proceed with spinal anaesthesia.

Standard monitoring was applied. Intravenous access was secured, and the patient was preloaded with 500 mL of Voluven. Spinal anaesthesia was performed in the sitting position at the lumbar level of L4/L5 using aseptic technique. Hyperbaric bupivacaine 0.5% (2 mL) combined with fentanyl 10 µg was administered intrathecally after confirmation of free cerebrospinal fluid flow. Sensory blockade to the T4 dermatome was achieved.

Intraoperative hypotension was anticipated, and phenylephrine was administered by repeated small aliquots titrated to effect. The recorded cumulative dose of 3 mg reflects total phenylephrine administered across the case (including syringe titration/infusion equivalent) rather than discrete boluses. A total of 1000 mL of Ringer’s lactate was infused intraoperatively. Baseline blood pressure was 120/80 mmHg (MAP 100) with an average intraoperative heart rate of 75-80 bpm; the lowest recorded blood pressure was 95/50 mmHg (MAP 65). Haemodynamic stability was maintained throughout the procedure, and blood pressure in recovery was 105/58 mmHg (MAP 73). Estimated blood loss was approximately 400 mL.

The caesarean section proceeded uneventfully, resulting in the delivery of a healthy neonate with Apgar scores of 9 at one minute and 10 at five minutes. The patient remained comfortable throughout surgery. Postoperatively, she was transferred to the recovery unit and subsequently to the ward. Neurological examination remained normal, and no complications related to the neuraxial block were observed.

## Discussion

This case illustrates the successful use of spinal anaesthesia for emergency caesarean delivery in a parturient with previously undiagnosed neurofibromatosis of uncertain subtype, in the absence of preoperative neuroimaging. Anaesthetic management in such cases is inherently complex, requiring careful balance between theoretical risks and the practical realities of obstetric urgency, diagnostic uncertainty, and resource limitation. Anaesthetic decision-making in this case was guided by phenotype-based risk stratification, prioritising clinical neurological assessment over unavailable radiological classification.

Neurofibromatosis is a heterogeneous disorder with marked phenotypic variability. NF1 is significantly more common than NF2 and is predominantly characterised by cutaneous manifestations, while NF2 is defined by frequent central nervous system involvement, including spinal tumours and bilateral vestibular schwannomas [2,3]. On examination, these distinctions are critical for anaesthetic risk stratification, yet may not be readily available in patients who present without prior diagnosis or investigation.

In the present case, the patient had extensive cutaneous neurofibromas but no neurological symptoms, no focal deficits, and no features suggestive of raised intracranial pressure. Available population-based data suggest that clinically significant spinal tumours in NF1 are uncommon and, when present, are usually associated with progressive neurological symptoms such as radicular pain, motor weakness, or sphincter disturbance [2,3]. Conversely, central nervous system involvement in NF2 is typically symptomatic [3]. Based on this, in the absence of neurological features, the likelihood of high-risk spinal pathology was therefore considered low, despite the inability to formally classify the disorder.

The principal concern regarding neuraxial anaesthesia in neurofibromatosis is the theoretical risk of neurological injury or haemorrhage related to unrecognised spinal tumours or meningeal involvement [4]. However, evidence supporting routine avoidance of neuraxial techniques is limited and largely theoretical. Several case reports describe successful spinal or epidural anaesthesia in parturients with neurofibromatosis when neurological examination is normal [4,8].

Of particular relevance is the report by Lee et al., who described spinal anaesthesia for emergency caesarean delivery in a neurologically asymptomatic parturient with neurofibromatosis, in whom preoperative imaging was unavailable [9]. In that case, neuraxial anaesthesia was undertaken based on clinical assessment despite the inability to exclude spinal pathology radiologically, with an uncomplicated outcome. The parallels with the present case are notable, particularly with respect to the emergency context, lack of imaging, and reliance on phenotype-based decision-making.

The relationship between intracranial pathology and neuraxial anaesthesia has been clarified in contemporary reviews, which emphasise that raised intracranial pressure and impaired cerebrospinal fluid dynamics-rather than intracranial lesions alone-determine the risk of neurological deterioration following dural puncture [7]. In patients without clinical features of raised intracranial pressure, neuraxial anaesthesia may be safely performed [7]. This conceptual framework suggests a symptom-based approach in suspected neurofibromatosis when imaging is unavailable.

General anaesthesia is often perceived as the safer alternative in neurofibromatosis; however, it carries substantial risks. Airway management may be complicated by neurofibromas involving the tongue, pharynx, or larynx, as well as by cervical spine abnormalities and scoliosis [4]. Mediastinal neurofibromas, although uncommon, have been reported and may cause airway compression or restrictive lung disease [8]. In addition, neurofibromatosis is associated with pheochromocytoma, with reported prevalence up to 5.7%, and undiagnosed disease may precipitate catastrophic haemodynamic instability during induction or surgical stimulation [6].

These risks are particularly relevant in obstetric anaesthesia, where general anaesthesia is independently associated with increased maternal morbidity and mortality compared with neuraxial techniques [7]. Avoidance of airway instrumentation and improved haemodynamic control are well-recognised advantages of neuraxial anaesthesia in caesarean delivery. These considerations were central to the anaesthetic strategy in both the present case and that reported by Lee et al. [9].

The resource-limited setting in which this case occurred further influenced decision-making. In many healthcare environments, access to urgent magnetic resonance imaging, genetic testing, or subspecialist consultation is limited. Requiring definitive classification or imaging before considering neuraxial anaesthesia in all patients with suspected neurofibromatosis would likely increase reliance on general anaesthesia or delay emergency delivery, without clear evidence of improved safety [4,8,9].

Spinal anaesthesia was performed with anticipation of hypotension secondary to sympathetic blockade. Phenylephrine was used to maintain maternal blood pressure, consistent with evidence supporting its efficacy in preserving uteroplacental perfusion during spinal anaesthesia for caesarean delivery [7]. Haemodynamic stability was maintained, and no adverse maternal or neonatal outcomes were observed.

In summary, this case supports a pragmatic, phenotype-based approach to anaesthetic decision-making in parturients with suspected but unclassified neurofibromatosis. When neurological examination is normal, and features suggestive of raised intracranial pressure or catecholamine excess are absent, spinal anaesthesia may be a safe and effective option, even in emergency and resource-limited settings. Careful documentation, informed consent, and preparedness for rapid conversion to general anaesthesia remain essential. However, this report is limited by the absence of preoperative imaging, genetic classification and long-term neurological follow-up.

## Conclusions

This case illustrates the challenges of anaesthetic decision-making in a parturient with previously undiagnosed neurofibromatosis of uncertain subtype requiring emergency caesarean delivery in a resource-limited setting. Particularly in emergency obstetric contexts where access to neuroimaging is limited. In the absence of prior investigations or neuroimaging, management relied on careful clinical assessment, focused neurological examination, and phenotype-based risk stratification. Despite theoretical concerns, spinal anaesthesia was safely performed without immediate maternal or neonatal complications.

This case illustrates the feasibility of a pragmatic, clinically driven approach whereby neuraxial anaesthesia may be considered when there are no neurological symptoms or signs of raised intracranial pressure or catecholamine excess, even when definitive classification and imaging are unavailable. Avoidance of general anaesthesia may mitigate airway and haemodynamic risks associated with neurofibromatosis, particularly in emergency obstetric contexts. This report reinforces the importance of senior input, informed consent, and preparedness to convert to general anaesthesia should clinical circumstances change.

## References

[1] Friedman JM (2002). Neurofibromatosis 1: clinical manifestations and diagnostic criteria. J Child Neurol.

[2] Huson SM, Harper PS, Compston DA (1988). Von Recklinghausen neurofibromatosis. A clinical and population study in south-east Wales. Brain.

[3] Fox CJ, Tomajian S, Kaye AJ, Russo S, Abadie JV, Kaye AD (2012). Perioperative management of neurofibromatosis type 1. Ochsner J.

[4] Hirsch NP, Murphy A, Radcliffe JJ (2001). Neurofibromatosis: clinical presentations and anaesthetic implications. Br J Anaesth.

[5] Terry AR, Barker FG 2nd, Leffert L, Bateman BT, Souter I, Plotkin SR (2013). Neurofibromatosis type 1 and pregnancy complications: a population-based study. Am J Obstet Gynecol.

[6] Walther MM, Herring J, Enquist E, Keiser HR, Linehan WM (1999). Von Recklinghausen’s disease and pheochromocytomas. J Urol.

[7] Leffert LR, Schwamm LH (2013). Neuraxial anesthesia in parturients with intracranial pathology: a comprehensive review and reassessment of risk. Anesthesiology.

[8] Dounas M, Mercier FJ, Lhuissier C, Benhamou D (1995). Epidural analgesia for labour in a parturient with neurofibromatosis. Can J Anaesth.

[9] Lee WY, Shin YS, Lim CS, Chung WS, Kim BM (2013). Spinal anesthesia for emergency cesarean section in a preeclampsia patient diagnosed with type 1 neurofibromatosis. Korean J Anesthesiol.

